# Effects of Electromigration on Sn-Bi Lead-Free Solder Alloy Joints on Copper and Copper with Nickel Surface Finish

**DOI:** 10.3390/ma18163722

**Published:** 2025-08-08

**Authors:** Lohgaindran Jeyeselan, Ervina Efzan Mhd Noor

**Affiliations:** 1 Centre for Manufacturing, Environment and Sustainability (CMES), CoE Robotics & Sensing Technologies, Multimedia University, Ayer Keroh, Melaka 75450, Malaysia; lohgain04@gmail.com; 2 Faculty of Engineering and Technology, Multimedia University, Ayer Keroh, Melaka 75450, Malaysia

**Keywords:** solders, lead-free, elements, electromigration, Sn-Bi, copper, substrate

## Abstract

Electromigration (EM) is a critical reliability concern in electronic solder joints due to increasing current densities in modern electronic packaging. EM-induced failures often manifest as void formation and microstructural degradation, particularly at the cathode interface. To address this issue, composite solder joints with elemental additions have been explored to enhance performance under high current stress. This study investigates the effect of Zn addition on the electromigration behavior and mechanical performance of eutectic Sn-Bi solder joints on copper (Cu) and nickel-coated copper (Ni/Cu) substrates. The solder alloys 58Sn-42Bi and Zn-modified Sn-Bi were prepared and reflowed onto the substrates. Electromigration testing was performed under a constant current of 1000 mA at room temperature, with applied voltages of 5 V, 12 V, and 24 V over a 10-day period per sample. Shear tests were conducted at a crosshead speed of 0.1 mm/min to evaluate joint strength. The results revealed that Zn addition influenced the distribution of Bi within the solder matrix, reducing Bi depletion at the cathode and mitigating accumulation at the anode, suggesting improved EM resistance. Zn-containing solder joints also demonstrated enhanced shear strength compared to unmodified Sn-Bi joints. These findings highlight the potential of Zn as a beneficial alloying element for improving the reliability of lead-free solder joints and form a foundation for future studies incorporating phase analysis and predictive EM lifetime modelling.

## 1. Introduction

Soldering can be characterized on the basis of the joining together of two materials without applying anything else [1]. Sn-Pb solders, which are thought of as traditional solders, have been widely applied in the field of electronic packaging for many decades. Concerning the toxicity of Pb to human health and the environment, many countries have limited or banned the production and application of Sn-Pb solders in many fields by means of legislation [2].

Until now, numerous examinations of lead-free solder material have been analyzed by researchers to discover alternatives. However, numerous materials are not accessible at some reflowing or working conditions, although the number of candidates is enormous, because these days the temperature of the reflowing procedure is lower than that of the past. In this manner, even though materials with a high melting point such as Sn–Ag–Cu are significantly welcome in the market, they cannot be implemented in some exceptional cases, particularly when the solder size is extremely small [3].

On the other hand, among these most favorable lead-free solder alloys, the Sn-Bi solder alloys have better properties, for example, a lower liquefying temperature, high tensile strength, good reliability, and excellent creep resistance. At the same time, the cost of Sn-Bi solder is lower than others [2]. The low melting point and coefficient of thermal expansion (CTE) can be differentiated to a eutectic Sn-Pb alloy, and the eutectic Sn-Bi alloy (Sn–58.1 wt% Bi) is a standout amongst the higher possible substitutions and it is basically prescribed to be utilized as part of temperature-sensitive processes [4].

However, beyond the issue of the melting point, numerous other challenges must be considered. A greater number of failure mechanisms can significantly reduce the performance of solder balls or even directly lead to complete product failure. Intermetallic compounds (IMCs) such as Cu_6_Sn_5_ and Ni_3_Sn_4_ typically form at the solder/substrate interface. Under electromigration (EM) conditions, these IMCs may grow unevenly or detach due to atomic flux, contributing to interfacial degradation. The interface thus becomes a critical failure point, exhibiting cracking, void formation, or thinning of the solder layer. Consequently, electromigration has become one of the most critical reliability challenges in solder joints [5]. In Sn-Bi-based solder joints subjected to current stressing, Bi atoms migrate in the direction of electron flow and accumulate at the anode. Furthermore, studies have shown that the diffusion behavior of Bi atoms can be altered through the addition of certain alloying elements, which helps reduce Bi segregation [6]. The advantages of utilizing a eutectic Sn-Bi solder alloy as a target element to concentrate on with respect to electromigration behavior is attributable to its common lamella microstructure subsequent to the 1st reflow. The Sn-rich phase and Bi-rich phase tend to isolate against each other after electromigration. In this way, the impact of electromigration can be assessed by the estimation of the aggregate layer of the Bi-rich phase at the anode interface. It can be said that the thinner the Bi-rich layer, the greater the electromigration resistance [7].

The eutectic Sn-Bi solder has an unstable microstructure; extreme stage coarsening will happen under thermal aging. Adding a small quantity of Zn into the solder is suggested to make the solder more stable. Hence, a recent review demonstrates that Zn addition could improve the electromigration impacts and lead to more Bi flux on the anode side [8]. Moreover, the suppression of atomic migration and control of diffusion paths, much like Zn’s role in Sn-Bi solders, has also been demonstrated in bilayer oxide systems and polymer-dispersed smart materials [8,9]. In current microelectronic innovation, soldering continues to play a crucial role. In the soldering filler metals, solders with liquefying focuses underneath 425 °C are accepted [10,11]. In this paper, testing will be done to study the effect of electromigration on Sn-Bi and Sn-Bi-X lead-free solder alloy joints on copper with a nickel surface finish.

## 2. Methodology

Material selection is a critical step in identifying the most suitable solder alloy for potential industrial applications. In this study, two lead-free solder alloys, 58Sn-Bi and Sn-9Zn-Bi, were prepared by melting the respective alloying elements at 450 °C for 1 h in a graphite crucible to ensure homogeneity. The solidified solder was then shaped into cylindrical billets with a diameter of 5 mm and a thickness of 1 mm.

For the shear test specimens, the solder billets were reflowed onto copper (Cu) and nickel (Ni) substrates (dimension: 40 mm × 10 mm) using a laboratory hotplate. The reflow process was conducted at 250 °C for 60 s to achieve proper metallurgical bonding, with the aid of a rosin-based flux to enhance wetting and remove surface oxides. The procedure was repeated for five samples to ensure consistency and reproducibility of the results.

For the electromigration test, a DC power supply was used to apply a constant current of 1000 mA, under three different voltages: 5, 12, and 24 V. The procedure was repeated for five samples for each condition to ensure consistency and reproducibility of the results. Each condition was maintained continuously for 10 days to study the electromigration effects in the solder joints, and maintained at room temperature (approximately 25 ± 2 °C) to observe the effects of electromigration on the solder joints. No pulsed current was applied during this test.

The mechanical properties of the solder joints were evaluated through shear testing using a crosshead speed of 0.1 mm/min. All tests were carried out under ambient laboratory conditions. The procedure was repeated for five samples to ensure consistency and reproducibility of the results.

After the tests were completed, the specimens were examined using an optical microscope at magnifications of 50 and 100×. Subsequently, the specimens were also examined using scanning electron microscopy (SEM) for higher-resolution analysis.

## 3. Results and Discussion

### 3.1. Shear Properties

The shear test was carried out at room temperature (25 °C) after the electromigration test of the solder billets soldered on to copper substrates with a nickel surface finish with the prior application of flux for both Sn-Bi and Sn-Zn-Bi. The gauge length of the solder is 40 mm, which is 20 mm from both sides of the specimens. The specimens with 5, 12, and 24 V were pulled vertically from both ends. The shear tests were carried out at a crosshead speed of 0.1 mm/min.

**Figure 1:**
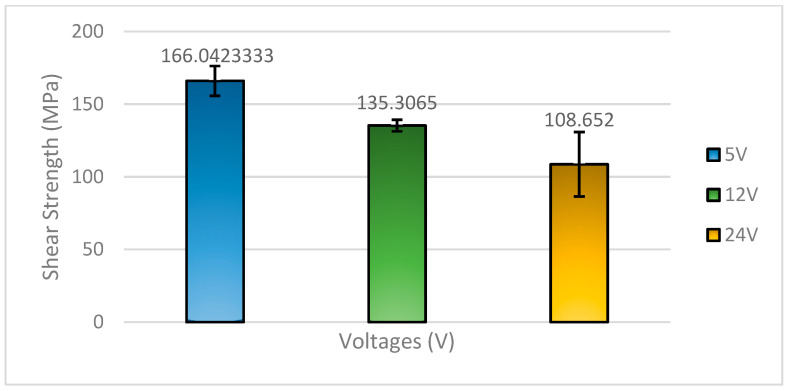
Shear strength of 58Sn-Bi solder with respect to flux applied.

Figure 1 shows the shear strength of the 58Sn-Bi solder alloy subjected to different voltage treatments. The results suggest that bismuth (Bi) atoms may accumulate at the anode side, leaving behind tin (Sn)-rich regions at the cathode, which could affect joint strength and uniformity. The results show that the application of 5 V produced the highest shear strength of 166.042 MPa, followed by 12 V at 135.3065 MPa, and 24 V at 108.652 MPa. This trend can be attributed to the effect of voltage on the microstructural evolution and interfacial reactions between the solder and the substrate material, which is typically copper (Cu) or nickel (Ni)-plated copper. At a moderate voltage of 5 V, the energy input is sufficient to promote uniform atomic diffusion and control intermetallic compound (IMC) formation at the solder/substrate interface. In particular, the formation of a thin, continuous Cu_6_Sn_5_ or (Ni,Cu)_3_Sn_4_ intermetallic layer enhances metallurgical bonding without compromising mechanical strength. The presence of bismuth (Bi), which is brittle, is also more finely distributed in the Sn-rich matrix under this condition, maintaining good joint ductility. However, as the voltage increases to 12 and 24 V, excessive joule heating and enhanced electromigration of Sn atoms towards the substrate can lead to the accelerated formation and growth of brittle IMC layers, such as Cu_3_Sn or thick Cu_6_Sn_5_. This is the same as stated by many researchers [12,13,14,15,16,17,18,19,20,21,22,23,24,25,26,27,28]. Simultaneously, bismuth atoms, which have limited solubility in Sn, may segregate and form coarse Bi-rich phases that act as crack initiation points under mechanical loading. This leads to the observed reduction in shear strength. At 24 V, the most significant degradation occurs, likely due to the formation of a thicker, brittle IMC layer and increased Bi segregation, weakening both the solder matrix and the solder/substrate interface.

**Figure 2:**
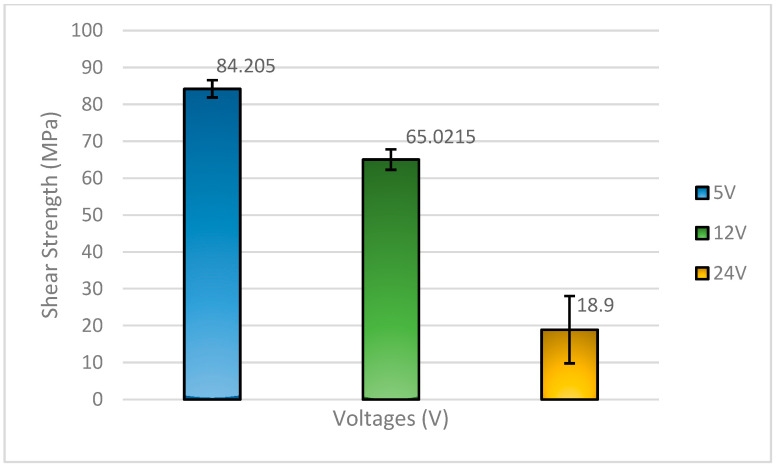
Shear strength of Sn-9Zn-Bi solder with respect to flux applied.

Figure 2 shows the shear strength comparison of the Sn-9Zn-Bi solder alloy under varying applied voltages. The results demonstrate that the solder treated with 5 V exhibited the highest shear strength at 84.205 MPa, followed by the 12 V-treated solder at 65.0215 MPa, and the 24 V-treated solder at a significantly lower value of 18.9 MPa.

This trend is closely related to the interactions between the solder alloy elements tin (Sn), zinc (Zn), and bismuth (Bi) and the substrate, typically copper (Cu) [14,15]. At 5 V, the electrical input is moderate, promoting controlled atomic diffusion and resulting in a more uniform microstructure. The presence of Zn in the solder enhances the formation of a thin and adherent Cu-Zn intermetallic layer (such as CuZn or Cu_5_Zn_8_) at the interface. These IMCs generally improve joint integrity due to their relatively better mechanical compatibility compared to thick, brittle Sn-Cu IMCs. Additionally, Bi acts as a solid solution strengthener and can refine the grain structure when well-distributed in the Sn matrix, thereby contributing to higher shear strength at a lower voltage.

As the applied voltage increases to 12 V, localized heating and enhanced atomic mobility accelerate the diffusion of Zn and Sn towards the Cu substrate. This leads to excessive growth of the IMCs, including brittle phases like CuZn_5_ or coarse Cu_5_Zn_8_, which can weaken the joint due to poor mechanical properties and stress concentration at the interface. Moreover, Bi may begin to segregate under higher thermal input, forming Bi-rich phases that are brittle and may initiate microcracks under shear stress.

At 24 V, the situation worsens significantly. High energy input may lead to excessive intermetallic compound (IMC) growth and the formation of voids, primarily due to rapid zinc (Zn) diffusion and electromigration effects. Zn has high reactivity with Cu and may deplete quickly from the solder matrix, leading to a Zn-depleted region with poor mechanical properties. Excess Bi segregation and the development of coarse, brittle Bi phases that further deteriorate the ductility and toughness of the joint. The combination of thick, brittle intermetallics and a weakened solder matrix explains the dramatic drop in shear strength to 18.9 MPa.

From previous works [8,14,15], it was concluded that the shear strength property of solders can be affected by the variation in reflow temperature and also as a function of Zinc (Zn). This study has also proven that the different voltages and different compositions of soldering also impact the shear strength of the solder with different results.

**Figure 3:**
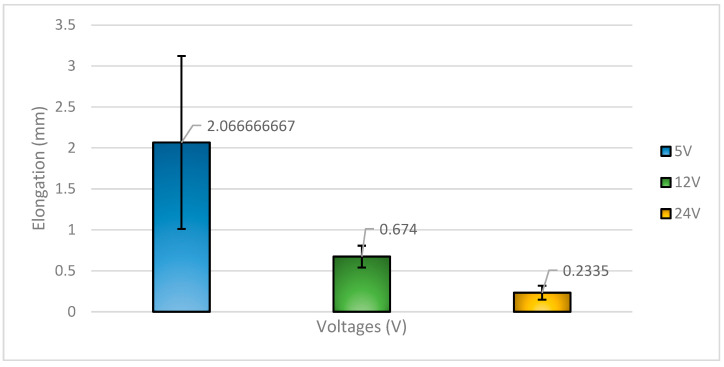
Elongation of 58Sn-Bi solder with respect to voltages applied.

In conjunction with the stress experienced by the specimens, the elongation data from Figure 3 provides further insights into the ductility of the 58Sn-Bi solder alloy under different voltage applications. The specimens subjected to 5 V demonstrated the highest elongation at 2.067 mm, followed by those treated with 12 V at 0.674 mm, and finally, the 24 V specimens at only 0.2335 mm. Notably, the 5 V-treated solder joint did not fracture even at the end of the shear test, indicating significant ductile behavior.

This enhanced elongation at 5 V suggests that the applied voltage provided just enough energy to promote uniform atomic diffusion and a more refined microstructure, while avoiding excessive intermetallic compound (IMC) growth or Bi segregation. The relatively soft and ductile Sn matrix remains continuous, and the Bi is better dispersed within the matrix at this voltage, maintaining a strong and tough microstructure. Additionally, at the solder/substrate interface, the formation of thin, controlled Cu-Sn IMCs (such as Cu_6_Sn_5_) contributes to good metallurgical bonding on the copper (Cu) or nickel-coated Cu without introducing excessive brittleness. In contrast, higher voltages, such as 12 V and especially 24 V, may lead to localized overheating and rapid electromigration, which promotes the growth of thick, brittle IMCs and the coarsening of Bi-rich phases. These brittle structures impede plastic deformation, reduce ductility, and act as crack initiation points during mechanical loading. Hence, the reduced elongation observed at these higher voltages reflects the degradation of both the solder bulk and the interfacial structure.

**Figure 4:**
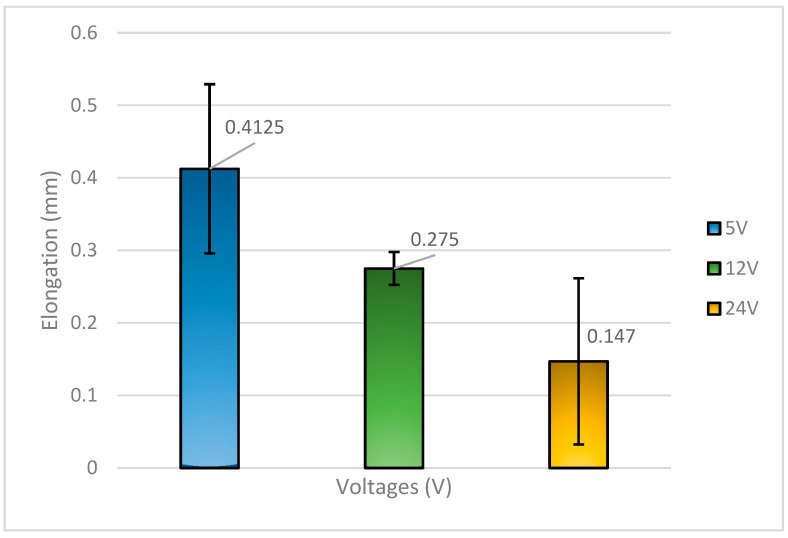
Elongation of Sn-9Zn-Bi solder with respect to voltages applied.

Figure 4 illustrates the elongation behavior of Sn-9Zn-Bi solder alloy billets subjected to different applied voltages. The solder specimens treated with 5 V exhibited the highest elongation at 0.413 mm, followed by those treated with 12 V at 0.275 mm, and finally, the 24 V specimens at 0.147 mm. These results clearly indicate that the solder with the highest shear strength also experienced the greatest elongation, while the one with the lowest shear strength corresponded to the lowest elongation, demonstrating a direct relationship between mechanical strength and ductility.

Although previous studies [2,18,20,21,22] have suggested that the addition of Zn to Sn-Bi alloys enhances their mechanical properties, the results from this work show a different outcome. Specifically, when the amount of Zn exceeds that of Bi, both the solder’s ductility and shear strength decline. This suggests that while moderate Zn additions can improve properties by forming strengthening intermetallic phases, excessive Zn leads to the formation of brittle Cu-Zn intermetallic compounds at the solder/substrate interface and alters the matrix composition unfavorably, resulting in a more brittle solder joint. Each voltage-treated solder exhibited some level of ductility; however, the degree of ductility diminished as the applied voltage increased. The most ductile behavior was observed at 5 V, where the electromigration effects were minimal, resulting in a more stable microstructure and controlled interfacial reactions. Conversely, the 24 V specimens, particularly for the Sn-9Zn-Bi alloy, not only withstood the least amount of stress but also showed the lowest elongation, confirming their brittle nature. This brittleness may be attributed to rapid Zn diffusion, thick, brittle intermetallic formation, and segregation effects under high voltage conditions.

Comparatively, the 58Sn-Bi solder treated at 5 V demonstrated superior mechanical performance, exhibiting both the highest tensile strength and the highest ductility among all tested conditions. This confirms the advantageous behavior of Sn-Bi at a lower voltage and without excess Zn, where Bi remains well-dispersed and IMC formation is optimally controlled.

**Figure 5:**
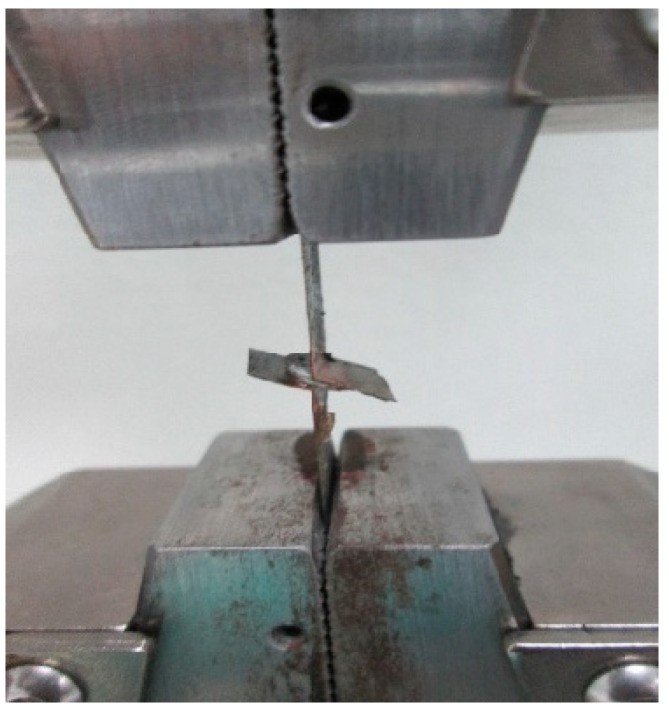
View of 58Sn-Bi (5 V) solder during shear test.

As shown in Figure 5, the view of the Sn-Bi solder at 5 V after the shear test confirms that the joint integrity remains intact, supporting the claim that this voltage level provides the most mechanically robust and ductile solder joint. Therefore, the 58Sn-Bi alloy treated at 5 V emerges as the most suitable choice for electrical or electronic applications where both strength and ductility are critical for long-term reliability.

Overall, the findings of this study confirm that increasing the voltage during the electromigration test leads to a higher likelihood of solder joint degradation and failure. Additionally, the mechanical properties of solder joints are highly sensitive to both alloy composition and applied electrical conditions. The careful selection of both solder composition and voltage parameters is therefore critical to optimizing solder joint performance in electronic packaging.

### 3.2. Microstructural Analysis

**Figure 6:**
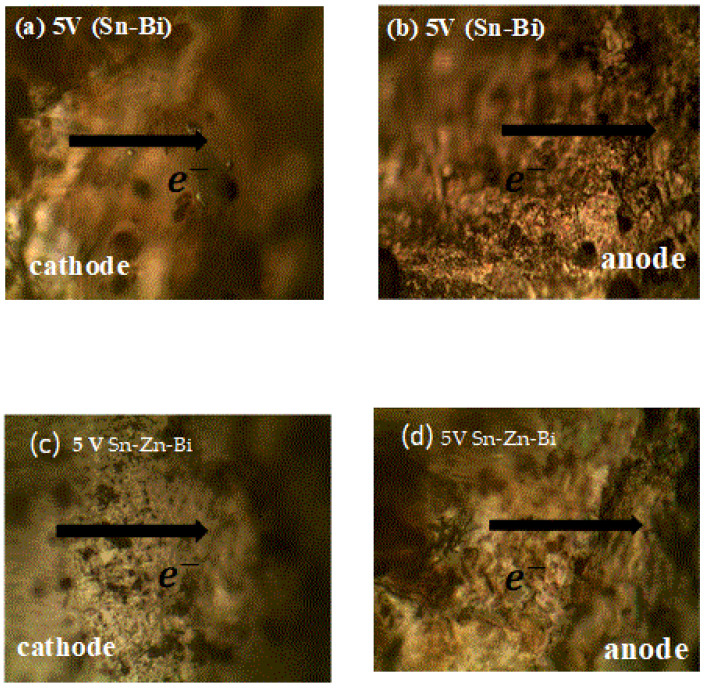
Shear test specimens for Sn-Bi and Sn-Zn-Bi for 5 V at 50× magnification.

The microstructures of the shear test specimens were observed after damage under an optical microscope. They were observed at a 50× magnification. As shown in Figure 6, the transfer of atoms from the cathode (left column) to anode (right column) for 5 V of Sn-Bi and Sn-Zn-Bi were observed. Figure 6a show that the amount of Sn and Bi atoms began to deplete at the cathode side and started to move towards the anode side, which can be seen in Figure 6a,c. As the voltage is small, the amount of transfer of the atoms from left to right is very small. Smaller dimples on its surface is indicative of damage from the shear test and can be seen at the anode side in Figure 6b,d. Zn can act as a barrier for the atom’s migration. Therefore, no clearer microstructural change can be seen.

**Figure 7:**
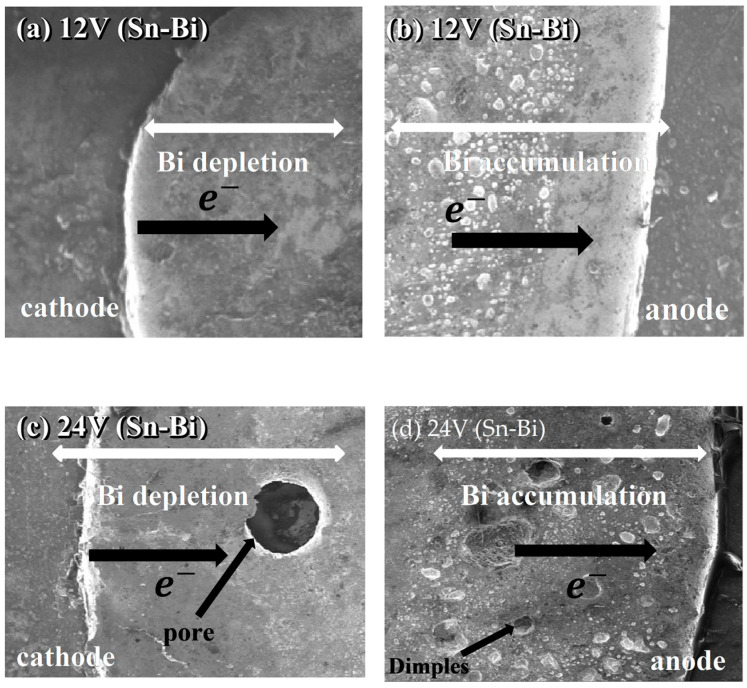
Shear test specimens for Sn-Bi for 12 V and 24 V at 20× magnification.

In this study, analyzing the microstructure of the elements used is influential for identifying the surface morphology observed through scanning electron microscopy (SEM). In this way, the formation of intermetallic compounds between the substrate and the solder is significant for analyzing the formed microstructure. Figure 7 shows the interaction between the solder and copper with the nickel substrate. Studies have shown that the interfacial reaction between solders and substrates that produce intermetallic compounds has a substantial effect on the mechanical properties and reliability of the solder joints [25]. These outcomes show the electromigration has an extreme influenced particularly on the cathode side of the joint. of the mechanical properties for solder joints [26,29,30,31,32].

### 3.3. Microstructural Analysis Through Scanning Electron Microscopy (SEM)

**Figure 8:**
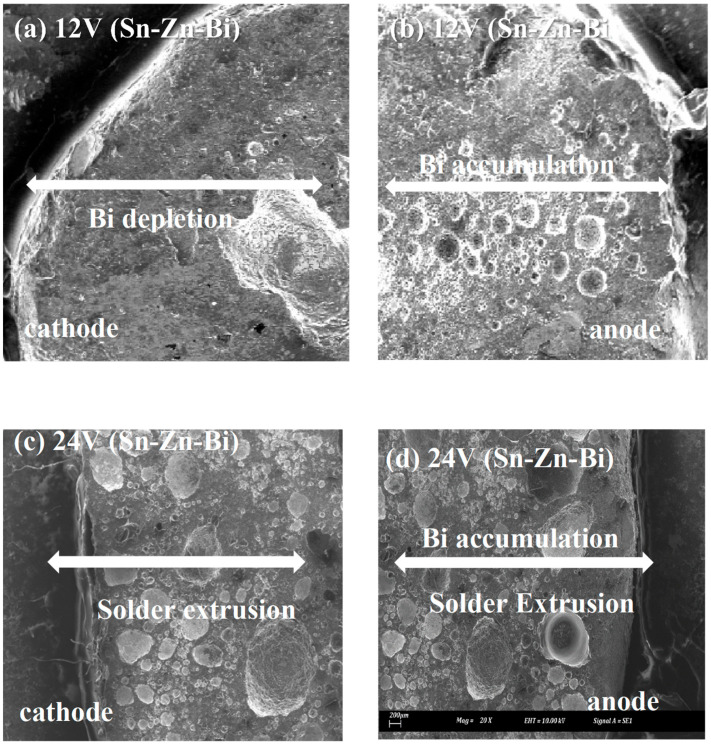
Shear test specimens for Sn-Zn-Bi for 12 V and 24 V at 20× magnification.

The microstructure of other specimens for 12 V and 24 V for were observed with scanning electron microscopy (SEM). They were observed at magnifications of 20× as shown in Figure 7 for 58Sn-Bi and in Figure 8 for Sn-9Zn-Bi. Figure 7a,c show the cathode part and Figure 7b,d show the anode part of the solder. When the voltages increase, the microstructure at both the cathode and anode side shows some variance. The solder depletes at the cathode while it is extruded at the anode part. The transfer of electrons from Sn and Bi from the cathode to anode part resulted in solder depletion at the cathode part. As a result, the solder atoms accumulate at the anode side. Figure 7a,c shows the images of the solder for 12 V and 24 V, and nearly complete Bi depletion at the cathode side.

These observations suggest that bismuth (Bi) migrates at a faster rate than tin (Sn) during the electromigration test. As a result, Bi atoms accumulate at the anode side, leaving behind Sn-rich regions at the cathode. This migration becomes more pronounced as the applied voltage increases. At 24 V, a large pore is observed at the left-hand side (anode), while numerous voids appear on the right-hand side (cathode). Due to the complex interplay of atomic movement and structural barriers, the amount of Bi leaving the cathode does not fully match the accumulation at the anode, resulting in a notable inconsistency between Bi depletion and accumulation across the solder joint.

A similar trend is observed in Figure 7, where the solder microstructures show surface defects, which are further highlighted in Figure 8. In Figure 8a–d, larger dimples and pores are evident at both the anode and cathode sides of the solder specimens, particularly in the samples subjected to higher voltages (12 V and 24 V). For the Sn-9Zn-Bi alloy, no clear Sn extrusion is visible at the anode side, implying that electromigration of Sn atoms is significantly suppressed under these conditions.

Moreover, the 12 V and 24 V samples show extensive void and pore formation on both sides of the solder joint, indicating severe degradation due to electromigration. This was also investigated by Li et al. [33]. For the 12 V Sn-9Zn-Bi specimen, Bi depletion is visible on the left side (cathode), while accumulation is observed on the right side (anode), consistent with directional Bi migration. This directional flow leads to localized mechanical and structural instability, contributing to void formation and embrittlement.

The solder microstructure remains relatively fine, which promotes a high grain boundary density. This microstructural characteristic facilitates greater Bi atom migration toward the anode due to enhanced grain boundary diffusion paths. While the addition of Zn is expected to act as a barrier to both Sn and Bi migrations, the experimental results show that when the Zn content exceeds that of Bi, it alters the migration dynamics. Specifically, higher Zn concentrations lead to excessive Bi accumulation across almost the entire solder joint, as clearly illustrated in Figure 8b,d. This behavior suggests that excessive Zn not only fails to sufficiently hinder Bi migration but may also contribute to undesirable redistribution of Bi, worsening the overall reliability of the solder joint under electromigration conditions.

**Figure 9:**
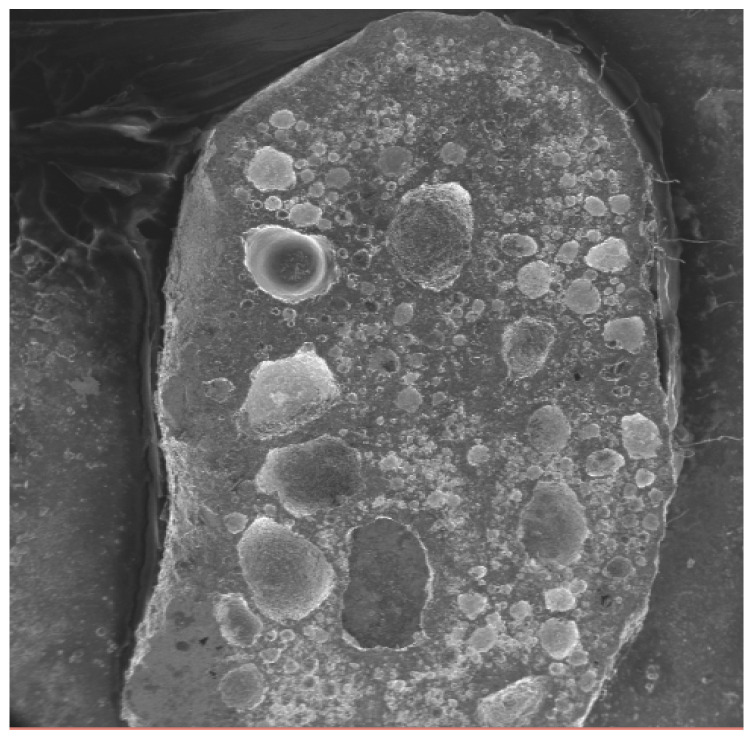
View of the solder of Sn-9Zn-Bi for 24 V.

Figure 9 shows the entire solder of Sn-9Zn-Bi solder at a magnification of 10×. The image retrieved from SEM clearly shows the amounts of voids, pores, and solder extrusion on the whole solder. Large dimples can be observed on the surface of the solder that used 24 V in Figure 8. In Figure 7b, smaller dimples are observed on the surface of the solder using 12 V, while solders using 5 V had the smallest dimples on their surface.

The solder that used small voltages had relatively smaller pores and dimples as compared to those that used a large voltage during electromigration tests. In Figure 6, the solder that uses 5 V clearly has minimal defects as compared to other voltages. Likewise, in Figure 8c,d, large voids are observed in solders in which 12 V was applied, followed by the soldiers that used 24 V, where too many pores and voids with higher accumulation of Bi atoms can be seen. This confirms once more that maximum ductility exists in solders that use 5 V, followed by 12 V, and then 24 V.

As stated earlier, CuNi/solder/CuNi specimens that used a lower voltage for joining were the most ductile, and the ductility seen with a lower voltage resulted in the solder undergoing minimal deformities in its structure, thus withstanding more stress over a longer period. The results revealed that electromigration has a significant impact on the Bi atoms that are able to migrate toward the anode side.

From this evaluation, it was seen that solders that were soldered with the composition of 58Sn-Bi possess the best mechanical properties and are the most ductile compared to Sn-9Zn-Bi. Soldering with the 58Sn-Bi for lower voltages would yield the best result for microelectronic applications, if adopted in the industry.

## 4. Conclusions

Electromigration tests were conducted on Sn-Bi and Sn-9Zn-Bi solder alloys under varying applied voltages, and their mechanical properties were assessed through shear testing. The shear tests were carried out to evaluate the alloys’ resistance to mechanical deformation and to identify the composition that offers superior performance under stress. Based on the results, Sn-Bi consistently exhibited higher shear strength compared to Sn-9Zn-Bi, indicating that Sn-Bi possesses better resistance to indentation and is mechanically more robust.

Microstructural analysis of the shear-tested specimens further supported this observation. Specimens exposed to 24 V exhibited the most severe structural defects, such as pores and cracks, followed by those treated with 12 V, and finally those at 5 V, which showed the least damage. Among all tested samples, Sn-Bi specimens subjected to 5 V showed the best mechanical behavior, exhibiting both high strength and ductility. In contrast, Sn-9Zn-Bi specimens tested at 24 V were the weakest and most brittle, with extensive voids and microstructural degradation.

These findings highlight two critical factors influencing solder performance under electromigration: the composition of the solder alloy and the magnitude of the applied voltage. The formation of intermetallic compounds (IMCs) at the solder/substrate interface plays a vital role in determining mechanical stability and electromigration resistance. In this study, a nickel (Ni) surface finish was employed on the copper substrate, serving as an effective diffusion barrier and enhancing the solderability of the joint. As the voltage increases, atomic migration from the cathode to anode is accelerated in both solder systems, resulting in greater Bi and Sn redistribution, increased void formation, and structural instability.

Although the addition of Zn in the Sn-Bi system is theoretically expected to hinder atom migration by blocking fast diffusion pathways, especially along grain boundaries. This effect is significantly diminished when the Zn content exceeds that of Bi. An imbalance in composition leads to poor phase distribution and can promote the excessive accumulation of Bi throughout the solder, thereby reducing the overall mechanical performance and reliability. In conclusion, the Sn-58Bi solder composition under low voltage conditions (particularly at 5 V) demonstrates superior electromigration resistance and mechanical strength compared to the Sn-9Zn-Bi alloy. These results suggest that careful control of both the solder composition and operational voltage is crucial in designing reliable solder joints for electronic applications.

Future research should explore the effects of alternative alloying elements such as antimony (Sb) and selected rare earth elements, which may offer improved thermal stability and microstructural refinement. In addition, integrating the experimental results with predictive models such as Black’s equation could provide a quantitative framework for estimating electromigration lifetime, thereby enhancing the reliability assessment of next-generation solder materials in real-world operating environments.

## Data Availability

The original contributions presented in this study are included in the article. Further inquiries can be directed to the corresponding author.
